# Supplementary material for: Verification of the Interaction Target Protein of the Effector ApCE22 of Arthrinium phaeospermum in Bambusa pervariabilis × Dendrocalamopsis grandis
Source: Biomolecules. 2023 Mar 25;13(4):590. doi: 10.3390/biom13040590 (PMC10135459; doi:10.3390/biom13040590)
Supplement: Supplementary file 1 [file biomolecules-13-00590-s001.zip › biomolecules-2214818-supplementary.pdf]

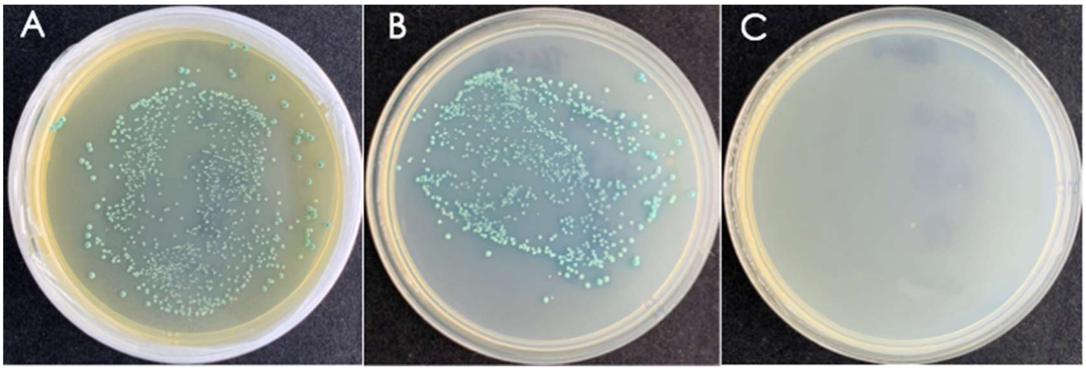

Figure S1. Self-activation detection of pGBKT7–ApCE22. Note: A: The reaction of the pGBKT7–ApCE22 bait and pGADT7-T was spread on the SD/-Leu/-Trp/X-α-gal medium. B: The reaction of the pGBKT7–ApCE22 bait and pGADT7-T was spread on the SD/-Leu/-Trp/-His/X-α-gal medium. C: The reaction of the pGBKT7–ApCE22 bait and pGADT7-T was spread on the SD/-Leu/-Trp/-His/-Ade/X-α-gal/AbA medium

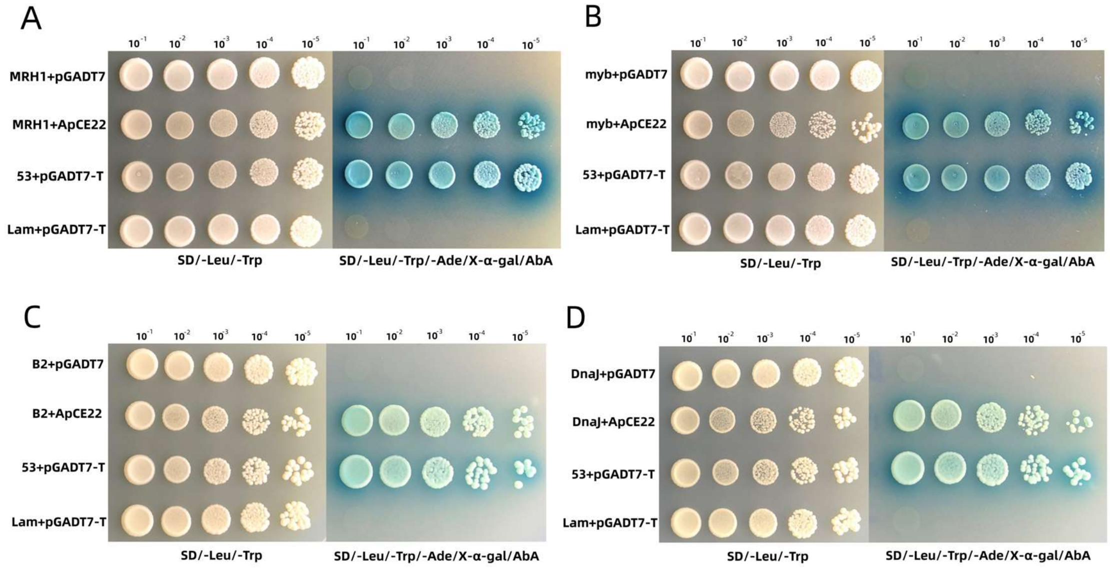

Figure S2. One-to-one yeast two-hybrid results of ApCE22 and the interacting proteins. Note: Interaction proteins were as a bait protein, and ApCE22 protein was as a prey protein.

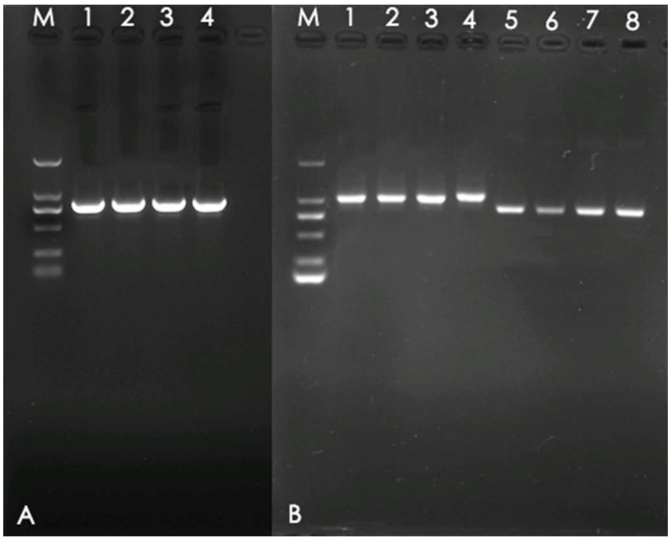

Figure S3. The gene detection results of recombinant plasmid pSPYNE(R) 173-ApCE22, pSPYCE(M)-B2 and pSPYCE(M)-DnaJ. M: DL2000 DNA marker, The labels of the ladder from top to bottom represent 2000, 1000, 750, 500, 250 and 100bp, respectively; 1, 2, 3, 4: ApCE22 gene of pSPYNE(R) 173-ApCE22 was detected by primer ApCE22-F/R; B: pSPYCE(M)-B2. M: DL2000 DNA marker, The labels of the ladder from top to bottom represent 2000, 1000, 750, 500, 250 and 100bp, respectively; 1, 2, 3, 4: B2 gene of pSPYCE(M)-B2 was detected by primer B2-F/R. 5, 6, 7, 8: DnaJ gene of pSPYCE(M)-DnaJ was detected by primer DnaJ-F/R.

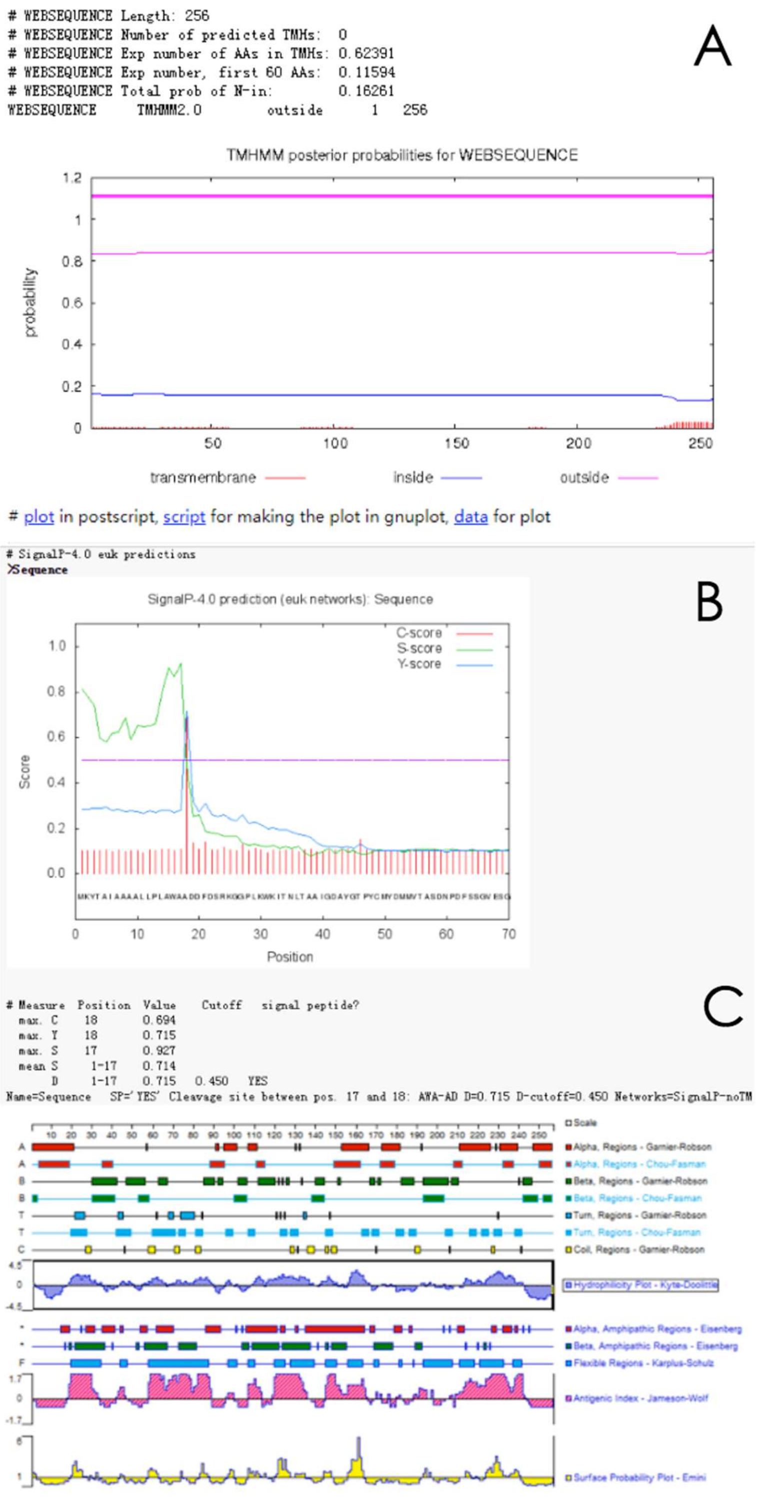

Figure S4. Analysis of transmembrane region, signal peptide and hydrophilicity of effector ApCE22 sequence

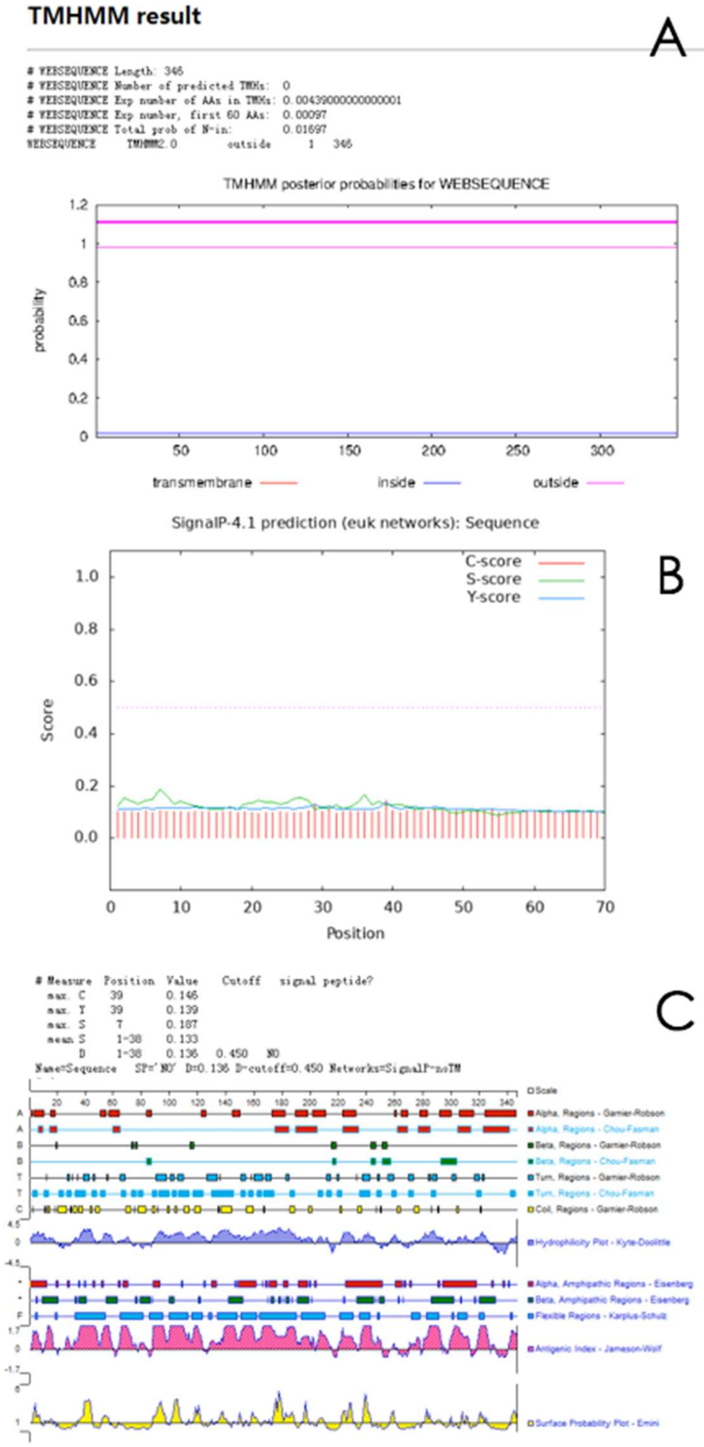

Figure S5. Analysis of transmembrane region, signal peptide and hydrophilicity of effector B2 sequence

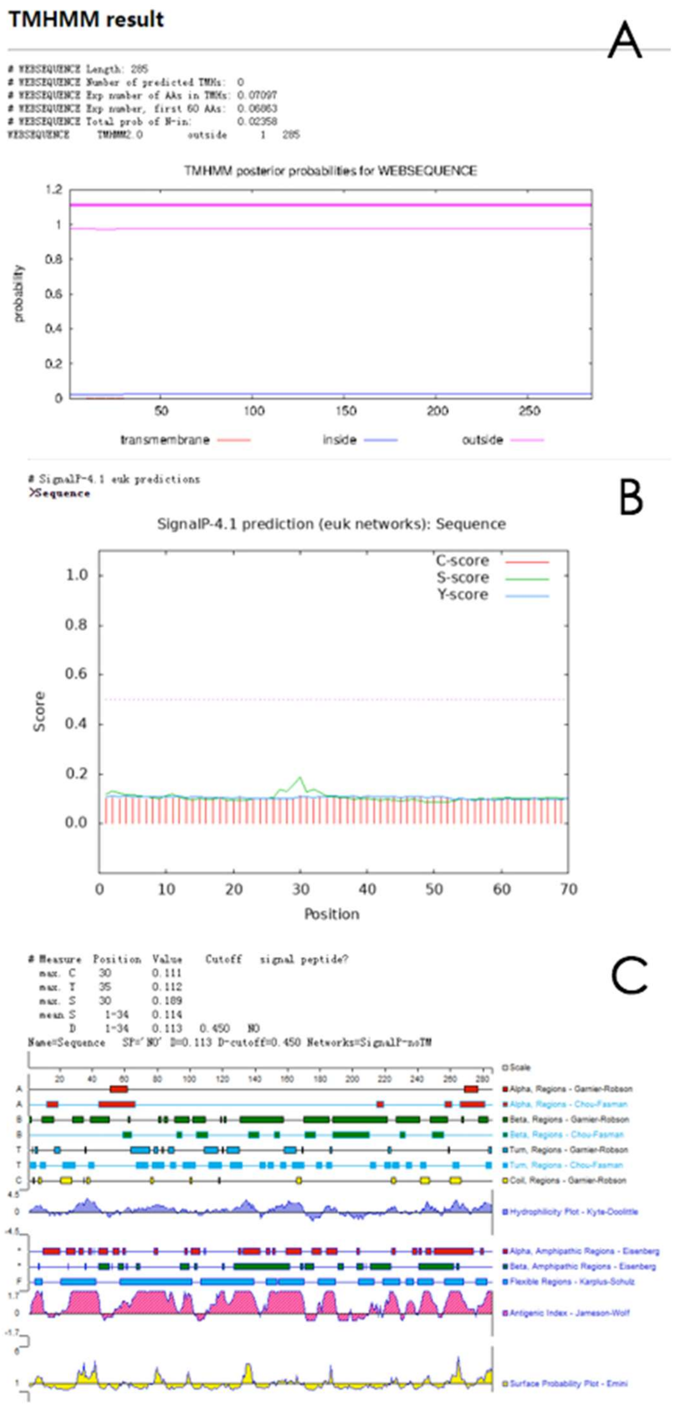

Figure S6. Analysis of transmembrane region, signal peptide and hydrophilicity of effector ChaJA6 sequence

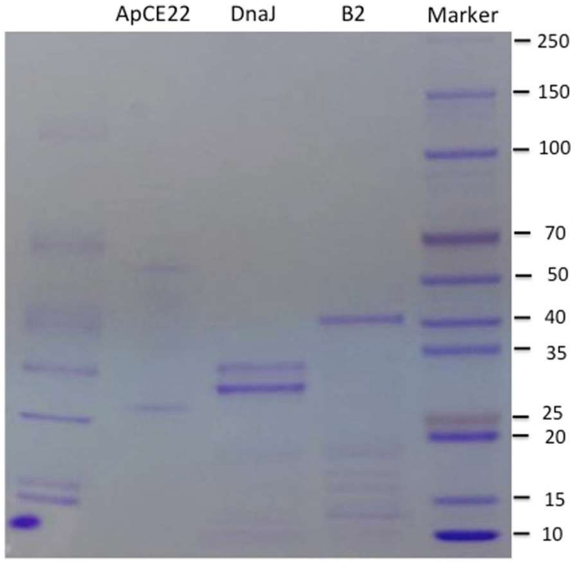

Figure S7. Electrophoresis results of the purified protein of the effector ApCE22, and the target proteins B2 and DnaJ. M: DL250KD protein marker, 1: ApCE22 protein; 2: DnaJ protein; 3: B2 protein.

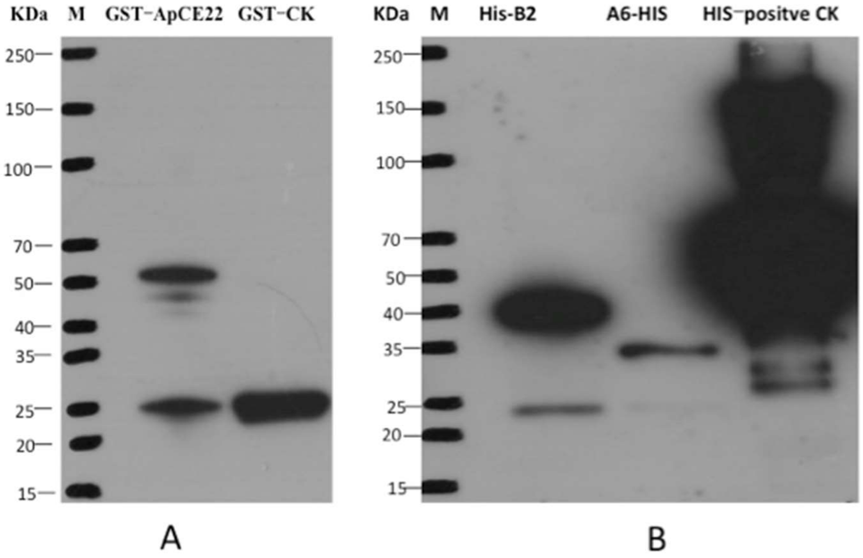

Figure S8. Western blot results of the effector ApCE22, the B2 protein and the DnaJ protein. Note: A: ApCE22 protein. B: B2 protein and DnaJ protein. M: DL250KD protein marker.

Table S1. Primer sequences of various expression vectors

Primer name	Primer sequence 5′-3′
pGBKT7-B2-F	AGGCCGAATTCCCGGGGATCC ATGGAGGGATACGACCGCGAGT
pGBKT7-B2-R	CCGCTGCAGGTCGACGGATCC TCAGGCATCATCCTTGTCTGCAAAGAT
pGBKT7- DnaJ-F	AGGCCGAATTCCCGGGGATCC ATGGGCACAGGAGATTACTCAAACCCGT
pGBKT7- DnaJ -R	CCGCTGCAGGTCGACGGATCC TCATCTCCTGCTATTGGCTGTTTGAGCCT
pGBKT7-MRH1-F	AGGCCGAATTCCCGGGGATCC ATGTCCCCGAGCTGGCCGG
pGBKT7-MEH1-R	CCGCTGCAGGTCGACGGATCC TCAGGTAGCTTCAGCAGACATGATC
pGBKT7-myb-F	AGGCCGAATTCCCGGGGATCC ATGGGGAGGGCGCCGTGCT
pGBKT7-myb-R	CCGCTGCAGGTCGACGGATCC TTAGCACGCGTCAGACAGGAGCCA
pGBKT7-ApCE22-F	AGGCCGAATTCCCGGGGATCCATGAAGTACACCGCGATCGC
pGBKT7-ApCE22-R	CCGCTGCAGGTCGACGGATCCTTAAAGGACCATAGCCATAAGACC
pGADT7-ApCE22-F	TGGGCATCGATACGGGATCC ATGAAGTACACCGCGATCGC
pGADT7-ApCE22-R	AGCTCGAGCTCGATGGATCC TTAAAGGACCATAGCCATAAGACC
pGADT7-B2-F	TGGGCATCGATACGGGATCC ATGGAGGGATACGACCGCGAGT
pGADT7-B2-R	AGCTCGAGCTCGATGGATCC TCAGGCATCATCCTTGTCTGCAAAGAT
pGADT7-DnaJ-F	TGGGCATCGATACGGGATCC ATGGGCACAGGAGATTACTCAAACCCGT
pGADT7- DnaJ 6-R	AGCTCGAGCTCGATGGATCC TCATCTCCTGCTATTGGCTGTTTGAGCCT
pGADT7-MRH1-F	TGGGCATCGATACGGGATCC ATGTCCCCGAGCTGGCCGG
pGADT7-MRH1-R	AGCTCGAGCTCGATGGATCC TCAGGTAGCTTCAGCAGACATGATC
pGADT7-myb-F	TGGGCATCGATACGGGATCC ATGGGGAGGGCGCCGTGCT
pGADT7-myb-R	AGCTCGAGCTCGATGGATCC TTAGCACGCGTCAGACAGGAGCCA
pSPYNE(R)173-ApCE22-F	GGGCCCAGGCCTACTAGTGGATCCATGAAGTACACCGCGATCGC
pSPYNE(R)173- ApCE22-R	GGTACCCTCGAGGTCGACGGATCCTTAAAGGACCATAGCCATAAGACC
pSPYCE(M)-B2-F	GCCTGGCGCGCCACTAGTGGATCCATGGAGGGATACGACCGCGAGT
pSPYCE(M)-B2-R	GTCGACAGTACTATCGATGGATCCTCAGGCATCATCCTTGTCTGCAAAGAT
pSPYCE(M)-DnaJ-F	GCCTGGCGCGCCACTAGTGGATCCATGGGCACAGGAGATTACTCAAACCCGT
pSPYCE(M)-DnaJ-R	GTCGACAGTACTATCGATGGATCCTCATCTCCTGCTATTGGCTGTTTGAGCCT
PGEX-6P-1-*ApCE22*-F	CAGGGGCCCCTGGGATCCATGAAGTACACCGCGATCGC
PGEX-6P-1- *ApCE22*-R	CGGGAATTCCGGGGATCCTTAAAGGACCATAGCCATAAGACC
pET28a-B2-F	CAAATGGGTCGCGGATCC ATGGAGGGATACGACCGCGAGT
pET28a-B2-R	GAGCTCGAATTCGGATCCTCAGGCATCATCCTTGTCTGCAAAGAT
pET28a-DnaJ-F	CAAATGGGTCGCGGATCC ATGGGCACAGGAGATTACTCAAACCCGT
pET28a-DnaJ-R	GAGCTCGAATTCGGATCCTCATCTCCTGCTATTGGCTGTTTGAGCCT

Note: the underline represents the upstream and downstream complementary sequence at the BamHI digestion site on the pGBKT7vector; the dashed underline represents the downstream complementary sequence at the upper and lower BamHI digestion sites of the pSPYNE(R)173 vector; the wavy lines represent the upstream and downstream complementary sequences at the BamHI digestion site on the pSPYCE(M) vector; the dot-dash underline represents the upstream and downstream complementary sequence at the BamHI digestion site on the PGEX-6P-1 vector; The bold underline represents the upstream and downstream complementary sequence at the BamHI digestion site on the pET28a vector. Double underscores represents the upstream and downstream complementary sequence at the BamHI digestion site on the pGADT7 vector.

Table S2. List of different plasmid ingredients in the self-activation and toxicity test.

Plasmid 1(100ng)	Plasmid 2（200ng）	Culture medium
pGBKT7-53	pGAT7-T	SD/-Trp/-Leu/X-a-gal
pGBKT7-Lam	pGAT7-T	SD/-Trp/-Leu/X-a-gal
pGBKT7–	pGAT7-T	SD/-Trp/-Leu/X-a-gal
ApCE12/ApCE22		SD/-Trp/-Leu/-His/X-a-gal
		SD/-Trp/-Leu/-His/-Ade/X-a-gal/AbA
pGBKT7		SD/-Trp
